# Preliminary esophageal microbiome studies prompt important scientific questions

**DOI:** 10.1038/s41424-018-0029-0

**Published:** 2018-05-29

**Authors:** Yash Choksi, Michael F. Vaezi

**Affiliations:** Division of Gastroenterology, Hepatology and Nutrition, Vanderbilt Medical Center, Nashville, TN USA

## Abstract

Analysis of the esophageal microbiome remains a new field of research. Two hypothesis-generating papers published in the current issue of the Journal go beyond characterizing the esophageal microbiome in Barrett’s esophagus or eosinophilic esophagitis (EoE). Snider et al. suggest that the salivary microbiome can be used as a screening tool for Barrett’s esophagus, and Arias et al. demonstrates abnormal expression of Toll-like receptors and innate immune effector proteins in patients with active EoE. We discuss these findings, raise fundamental questions about microbiome studies, and offer ideas for future studies.

Although it is known that the esophagus contains a diverse microbiome [1], analysis and interpretation of the microbiome in the context of esophageal disease is currently underexplored. Moreover, the rising incidence of Barrett’s esophagus (BE), esophageal adenocarcinoma (EAC), and EoE has caused some to ask whether shifts in the microbiome could be contributing. The average annual percentage increase in incidence of EAC was 6.1% in men and 5.9% in women from 1975 to 2009 [2] and is beyond what would be expected from increases in rates of GERD, obesity, and smoking [3]. Given these data, the widespread use of antibiotics starting in the 1940s, and the relatively stable incidence of EAC up until the 1960s [4], there is great interest in increasing our understanding of the esophageal microbiome in the context of EAC. Likewise, incidence of EoE has risen, especially in developed countries, to 4–400 per 100,000, in a similar time frame [5], prompting increased study of the role of the microbiome in the pathogenesis of EoE. Given these observations, these articles are timely. We feel that they provide important hypothesis-generating data upon which investigators can build.

In the first article, Snider et al. [6] demonstrate in a case-control study that the salivary microbiome, which has previously been shown to be similar but not identical to the esophageal microbiome [7, 8], can be used to accurately diagnose BE with high sensitivity (97%) and specificity (88%). Specifically, patients with BE had increased Firmicutes and decreased Proteobacteria, and there were numerous taxonomic differences in the oral microbiome between BE and controls, including relative abundance of *Lautropia, Streptococcus*, and a genus in the order *Bacteroidales*. In addition, patients with high-grade dysplasia or EAC had decreased *Veillonella* and increased *Enterobacteriaceae* as compared with those with non-dysplastic BE. Based on these results, the authors posit that the oral microbiome could potentially be used as a non-invasive screening tool for BE and potentially even for progression to EAC. Prior studies have focused on the esophageal microbiome. Yang et al. [9] compared the microbiomes of distal esophageal biopsies in 12 patients with normal appearance of the esophagus during endoscopy, 12 with esophagitis, and 10 with BE (none had dysplasia). Clustering analyses revealed that patients with BE or reflux esophagitis had a greater proportion of gram-negative anaerobes/microaerophiles as compared with controls. *Streptococcus* was found to be decreased in patients with BE  or with reflux esophagitis. In another study, MacFarlane et al. [10] cultured and sequenced esophageal biopsy and aspirate samples from seven patients with BE  and seven patients with a normal appearing esophagus on endoscopy. These authors showed high levels of *Campylobacter* in four of the seven patients with BE but in none of the control subjects.

While Snider et al. focused on the salivary microbiome rather than the esophageal microbiome, the range of results from prior publications illustrates that these preliminary studies should be interpreted with caution. A number of factors can affect the composition of the microbiome, including diet [11] and medications (e.g., proton pump inhibitors [12]). Future studies should attempt to control for these as best as possible, and these authors should be commended for this attempt. Likewise, further investigation into how the microbiome (both esophageal and salivary) can vary over time, even in someone without disease, is essential to improving understanding in how the microbiome mechanistically causes disease. These reports provide snapshots into a picture of the microbiome at a particular time but do not reveal whether the current microbiome is a result of the disease process or contributed to its beginning.

In the second article, Arias et al. [13] characterize the esophageal and duodenal innate immune response before and after dietary therapy in EoE patients. The authors establish that bacterial load and expression of Toll-like Receptors (TLRs), which are pattern-recognition receptors that recognize molecular patterns of microbes, are increased in patients with active EoE. Thus, in contrast to the first paper, these authors are focused on the consequences of an altered microbiome in disease pathogenesis rather than diagnosis. The authors show that there is increased bacterial load by 16S expression as well as increased expression of TLR1, TLR2, TLR4, and TLR9 in active EoE as compared with controls and those in clinical and histologic remission after treatment with the six food elimination diet (SFED). As bacteria can reside in the mucus layer, they next show that mucins (Muc1 and Muc5B) are downregulated in active disease, whereas Muc4, which is upregulated, was thought to be a compensatory increase. Expression of these mucins corrected to a level similar to controls after remission from SFED diet. Finally, the authors argue that upregulated TLRs have functional significance by demonstrating an upregulation of Myd88, the adapter protein all TLRs (except TLR3) interact with which activates nuclear factor-κB (NF-κB), in patients with active EoE. In support of this claim, they also show upregulation of NF-κB induced cytokines, such as IL-1β, IL-6, IL-8, and IL-10, upregulation of innate immune effector proteins PRF-1, iNOS, and GZMA, and upregulation of the NK-G2D system (i.e., IL-15, MICB, and KLRL1). All of these corrected after dietary intervention except for MICB. Notably, there was also increased expression of TLR1, TLR2, and TLR4 in non-inflamed duodenum of patients with active EoE. However, there was not increased bacterial load or upregulation of immune mediators.

As with the prior paper, these data raise a number of questions. First, in the discussion, the authors propose to define whether epithelial or immune cells are overexpressing TLRs. This is an excellent idea and may shed light on why tissue from the duodenum also shows dysregulated TLRs and which bacteria could be triggering inflammation. Second, prior findings have shown an increased relative abundance of *Neisseria* and *Corynebacterium* [14] (though in pediatric patients) or *Haemophilus* [15] (both in adult and pediatric patients) in active EoE versus controls. Can these particular bacteria result in the same change in TLRs found in this study? Third, does symptom duration or “time to evolution” correlate with the wide range of expression in TLRs from patient to patient? Does early EoE have a characteristic microbial composition versus patients with active disease for a long period of time vs. patients with stricturing disease?

In conclusion, the esophageal microbiome is poorly understood. The epidemiology of Barrett’s esophagus, EAC, and EoE suggest that the microbiome could be at least partially responsible for the rising incidence of all three conditions. The two papers published in the current issue are a great start at elucidating whether this hypothesis is correct. The first paper argues that the oral microbiome can accurately identify Barrett’s esophagus, whereas the second paper evaluates innate immune signaling in EoE and posits that this could be due to an altered microbiome. The first study could provide the basis for a non-invasive screening tool for BE, and the second study establishes that the microbiome has functional relevance in the pathogenesis of EoE. Although these findings are exciting and important for generating more research questions, they should be interpreted with caution. Both studies were done at a single center and in a small number of patients. Results from prior publications characterizing the microbiome of Barrett’s esophagus, EAC, and EoE exhibit varying results, and it is still unknown how most medications (including probiotics, antibiotics, anti-inflammatories) and environmental pressures (including diet) change the microbiome. These are fundamental questions that are essential to study design. Further, data regarding causality are lacking—most likely there are microbial changes that contribute to disease and other changes, which are the result of disease. These preliminary studies provide the evidence that prospective studies are needed to measure longitudinal changes in the microbiome in those with disease, those with remission, and those without disease. Such studies could both identify potential targets for risk modification, investigate the clinical utility of markers for disease risk, and help scientists and clinicians begin to understand the question of causality.
